# Evidence for the role of German final devoicing in pre-attentive speech processing: a mismatch negativity study

**DOI:** 10.3389/fpsyg.2014.01317

**Published:** 2014-11-25

**Authors:** Hubert Truckenbrodt, Johanna Steinberg, Thomas K. Jacobsen, Thomas Jacobsen

**Affiliations:** ^1^Centre for General LinguisticsBerlin, Germany; ^2^Institut für deutsche Sprache und Linguistik, Humboldt UniversityBerlin, Germany; ^3^Department of Psychology, University of LeipzigLeipzig, Germany; ^4^Experimental Psychology Unit, Helmut Schmidt University/University of the Federal Armed Forces HamburgHamburg, Germany

**Keywords:** mismatch negativity (MMN), event-related potentials (ERP), phonological rules, final devoicing, phonotactics, German, pre-attentive processing

## Abstract

Results of a mismatch negativity experiment are reported in which the pre-attentive relevance of the German phonological alternation of final devoicing (FD) is shown in two ways. The experiment employs pseudowords. (1) A deviant [vus] paired with standard /vuzə/ did not show a mismatch effect for the voicing change in /z/ versus [s] because the two can be related by FD. When standard and deviant were reversed, the two could not be related by FD and a mismatch effect for the voicing difference occurred. (2) An ill-formed deviant that violates FD, *[vuz], triggered mismatch effects that were plausibly attributed to its ill-formedness. The results show that a syllable-related process like FD is already taken into account by the processing system in early pre-attentive processing.

## INTRODUCTION

### NEURAL PROCESSING AND MISMATCH NEGATIVITY

Electrophysiological methods like the electroencephalogram (EEG) and the magnetoencephalogram (MEG) provide the possibility to obtain online insight into the perceptual process. This includes the pre-conscious and pre-attentive or automatic stages of processing. There is a sequence of positive–negative–positive deflections in the event-related potential (ERP), of which the first negative deflection (N100) typically peaks around 100 ms after the occurrence of a transient sound like an isolated vowel. The N100 has been found for speech sounds and for non-speech sounds. For speech sounds, the processing in this early stage shows, for one thing, characteristics of acoustic processing that are independent of phonological categories (e.g., Sharma and Dorman, 2000). At the same time, a number of studies have demonstrated the effect of phonological categories in this early stage: acoustically equidistant stimuli cluster along phonological categories. This can be observed in the exact timing of the effect ([a] at 95 ms, [u] at 120 ms; see Roberts et al., 2004), and in the location of the activity in the brain (Obleser et al., 2004).

The mismatch negativity (MMN) component of the ERP allows for some indirect insights into this early phase of processing. MMN is typically obtained in a classic passive oddball paradigm. In this experimental protocol, a sequence of identical sounds, the standards, (for example [a]), is interrupted occasionally by another sound, the deviant, (for example [u]), as in [a a a u ...]. A standard experimental design, called reversed oddball design, will test [a a a u ...] with standard [a] and deviant [u] as well as [u u u a ...] with standard [u] and deviant [a], both with a considerable number of repetitions. The activities of standard [u], deviant [u], standard [a], and deviant [a] are then each averaged separately, and the difference waves are calculated from the ERPs either by subtracting the ERP of the original standard from the deviant ERP or by subtracting the ERPs elicited by the same stimulus when presented as standard and as deviant from the reversed oddball condition. A significant negative-going deflection in the difference wave calculated from the deviant and the standard ERP may be evidence for the MMN ERP component (Näätänen, 1992, 2001). This often occurs in the time range of 100–250 ms after the beginning of the deviating sound (e.g., Schröger, 2005).

Mismatch negativity studies also show the early effect of phonological categories (see for example Dehaene-Lambertz et al., 2000; Phillips et al., 2000); the evidence for this comes in part from comparisons between speakers of different languages (Näätänen et al., 1997; Winkler et al., 1999; Peltola et al., 2003). The speakers may react differently to a given sound contrast depending on the sound inventory of their native language.

### PREVIOUS MMN-STUDIES ON PHONOTACTIC RESTRICTIONS

Some other studies have investigated the effects of phonological rules or phonotactic constraints in MMN protocols. Dehaene-Lambertz et al. (2000) investigated the Japanese restriction that the syllable coda allows only place-assimilated nasals (and the first part of a geminate; cf. Itô, 1986). When Japanese listeners hear a sequence like [igmo] they perceive the presence of an additional vowel as in [igumo]. The additional vowel makes the sequence well-formed in Japanese. French speakers do not hear such an additional vowel. In an MMN experiment, pairs like [igmo] and [igumo] were investigated for effects of the vowel epenthesis. In Japanese speakers, there was no MMN effect, while in French speakers there was. As the authors note, these results “suggest that the impact of phonotactics takes place early in speech processing and support models of speech perception, which postulate that the input signal is directly parsed into the native language phonological format” (p. 635). Since we were interested, in our own studies, in effects of processing that take place outside of the focus of attention, we mention that the participants of the study of Dehaene-Lambertz et al. (2000) were instructed to pay attention to the stimuli and to answer for each five-stimulus sequence whether the fifth (the deviant) was different from the preceding four.

Mitterer and Blomert (2003) investigated optional nasal place assimilation in Dutch compounds (in terms of lexical phonological theory: postlexical assimilation). They paired the unassimilated [tuinbank] with the assimilated [tuimbank] (both “garden bank”), both of which are possible forms of this word in Dutch, while participants were watching a silent movie. This contrast was compared with the pairing of [tuinstoel] and [tuimstoel]. While the difference between [tuin] and [tuim] was identical in the two stimulus pairs, the change was not motivated by assimilation in [tuimstoel]. A significant difference between standard and deviant was found in the latter pair, but not in the former pair where the assimilation process relates the two forms. Therefore, the regressive assimilation process is relevant to early pre-attentive processing.

Flagg et al. (2006) investigated an assimilatory nasalization process in English with an MEG study. In /ama/ the first vowel optionally gets nasalized by the following nasal as in [ãma]. Flagg et al. (2006) classified this alternation as phonological assimilation, though we point out that the process more likely is to be seen as coarticulatory, i.e., phonetic in nature. Such a nasalized vowel was spliced before a non-nasal consonant as in [ãba]. The participants of the experiment were watching silent movies during passive stimulation. A latency delay was found for the M50 response elicited by the incongruent plosive in [ãba] compared to [aba]. This indicated that the nasalization process was relevant to very early pre-attentive processing.

Steinberg et al. (2010a,b, 2011) investigated a German allophonic alternation related to two dorsal fricative allophones both represented orthographically as “ch.” The palatal allophone of this fricative occurs after front vowels ([dɪçt] *dicht* ‘dense’) and the velar allophone after back vowels ([dɔxt] *Docht* ‘wick’). This alternation is also known as dorsal fricative assimilation (DFA). From a range of different experiments that all provide evidence for the effect of DFA in pre-attentive processing, we here choose one for presentation: the ill-formed non-word ^∗^[εx] combines a velar fricative with a front vowel. Contrasted with the well-formed pseudoword [ɔx] as standard, there was a mismatch effect attributable to the different vowels. The fricatives are segmentally identical, so that the deviant [ɔx] did not show an MMN due to the fricative in the comparison condition. However, the ill-formed deviant ^∗^[εx] elicited an additional MMN response attributable to the fricative. This response was temporally separated from the vowel-related MMN and attributed to the abstract phonotactic ill-formedness of the deviant.

In the present study on final devoicing (FD) in German, we continue our investigation of bona fide productive lexical phonological rules in German, i.e., of alternations that apply obligatorily, without idiosyncratic exceptions and within the domain of words or pseudowords, but not across words or pseudowords.

### FINAL DEVOICING

Final devoicing operates on what has classically been analyzed as a voicing contrast (see e.g., Rubach, 1990; Hall, 1992). Jessen and Ringen (2002) have argued that the contrast instead involves the feature [spread glottis] for the plosives, and Beckman et al. (2009), building on this, have argued that the German fricatives are specified for both [spread glottis] and [voiced] (see also Vaux, 1998 for arguments that voiceless fricatives are specified [+spread glottis] across languages). In the present experiment, we employed a voicing distinction in fricatives. Assuming such a dual specification, we expect no effects of lexical underspecification, which have been argued to affect MMN by Eulitz and Lahiri (2004), Cornell et al. (2011, 2013), and Scharinger et al. (2012). Instead, voiced fricatives would be specified [+voiced] and voiceless ones would be specified [+spread glottis] in the mental lexical entries.

The German plosives and fricatives that allow such a laryngeal contrast, here transcribed in terms of voicing, are [p/b, t/d, k/g, f/v, s/z]. Both members of each pair can occur in the onset of a syllable before a vowel. In the classical analysis, the voiced values become voiceless in a syllable coda (Rubach, 1990; Hall, 1992). Thus, the two genitive forms [ra.d-əs] (*Rades* ‘wheel-GEN’) and [ra.t-əs] (*Rates* ‘advice-GEN’) distinguish [d] and [t] in the syllable onset before a vowel. However, in the nominative form, without the genitive suffix [-əs], the forms are identically pronounced [ra:t] (‘wheel’/‘advice’). Here, /d/ and /t/ are in the syllable coda and only the voiceless pronunciation [t] occurs. The change from /d/ in the mental lexical entry /rad/ to [t] in the pronunciation in coda position is called final devoicing (FD). There are different suggestions about the best way of describing and capturing the correct environment (see e.g., Lombardi, 1991, 1999; Steriade, 1997; Beckman et al., 2009). There is also a debate about whether the voicing neutralization is phonetically complete (see e.g., Port and O’Dell, 1985; Beckman et al., 2009). However, it is clear that the change takes place obligatorily in a set of core environments that include the word-final position, that there are no lexically marked exceptions, and that the change is bounded by the word.

Hwang et al. (2010) showed that English voicing agreement in consonant clusters as in their pseudoword stimuli [ʊts] and [ʊdz] lead to processing difficulties in non-agreeing clusters like ^∗^[ʊds], which were not found in non-agreeing clusters like ^∗^[ʊtz]. Poeppel and Monahan (2011, p. 947f.) refer to results of a related MEG experiment in which a distinction between [ʊts] and ^∗^[ʊds] was found around 150 ms after the onset of the fricative. Hwang et al. (2010) interpret their results in terms of the underspecification for voicing of voiceless plosives in English postulated by Lombardi (1991, 1999): speakers predict a following voiced sound after [d] in ^∗^[ʊds] but do not predict a following voiceless sound after [t] in ^∗^[ʊtz] because [t] is underspecified for voicing. We think that this explanation may apply to a phonological surface structure in which the position preceding the fricative is conceivably one of laryngeal neutralization (see Steriade, 1997). It is conceivable that the voicelessness of [t] preceding a fricative may be accounted for by laryngeal neutralization by the processing system, while the voicing of [d], if followed by a fricative, can only be licensed by agreement with the fricative. Our assumptions about the underlying featural specifications in German are thus not in conflict with these interesting results.

The voicing distinction between obstruents in German is phonetically implemented in several ways depending both on the manner of articulation and on the relative position of the sound. As we will focus on fricatives in intervocalic and final position in our study (see *Experimental design*), we will limit the following overview over phonetic voicing cues to these instances. Because in German, the phonetic implementations of the voicing contrast are – at least partly – neutralized in final positions, we also attend to voicing parameters obtained in languages like English in which FD is not operative. As shown by various phonetic studies (for an overview of the literature on the voicing distinction in German fricatives, see Jessen, 1998, pp. 65–66, 96; phonetic evidence on English fricatives is reviewed for instance by Stevens et al., 1992, and Maniwa and Jongman, 2009) the voicing distinction between fricatives is mainly coded by three kinds of parameters: first, the *duration of the fricative* (as reflected by the duration of friction noise in the acoustic signal) is shorter in voiced compared to voiceless fricatives. Preceding full vowels show – to some degree – the reversed durational pattern. Second, there are several spectral indicators for fricative voicing, most importantly the presence of periodic *low-frequency energy* during the fricative (reflecting vocal fold vibrations). Additionally, voiced fricatives are characterized by a lower Center of Gravity (COG) and higher variance compared to voiceless fricatives (cf. Maniwa and Jongman, 2009). Third, fricative voicing is indicated by a greater extent of the *F1 transitions* of preceding or following adjacent vowels (e.g., Stevens et al., 1992). Furthermore, vowels following a voiced fricative have been shown to begin with lower F_0_ than vowels after voiceless ones (Jessen, 1998).

### AN ASYMMETRY BETWEEN STANDARDS AND DEVIANTS

There is an interesting asymmetry in the roles played by standard and deviant in processing the oddball stimulation. Since the standard is repeated a number of times, and the pauses between the repetitions give sufficient time for it to be recognized as a particular phonological sound or sound sequence, it seems, put simply, that the expectation for another standard is phonologically represented, or represented in more abstract terms (Näätänen, 2001). The deviant, on the other hand, is just coming into the system and its initial processing is ongoing at the time when the mismatch against the standard arises.

Eulitz and Lahiri (2004), Cornell et al. (2011, 2013), and Scharinger et al. (2012) have argued that the standard can in certain ways be seen as similar to a mental lexical entry, likewise abstractly represented, and that the deviant can be seen as similar to the incoming acoustic information that the system seeks to match to an abstract lexical entry. They have argued that lexical underspecification of features matters for MMN in a way that can be understood in these terms. A crucial aspect of the asymmetry for our experiment is that it provides a direction of application of FD: if it applies in an oddball protocol in early pre-attentive processing, it should apply in pairs in which the standard corresponds to a possible mental lexical entry to which FD could apply, and in which the deviant can be seen as similar to a spoken word to which FD has applied. For ease of exposition we therefore adopt some notation of Cornell et al. (2013). The standards are provided with slashes /./ and the deviants with squared brackets [.]. This is parallel to the phonological notation where /./ is used for mental lexical entries and [.] for what is heard.

### EXPERIMENTAL DESIGN

**FIGURE 1:**
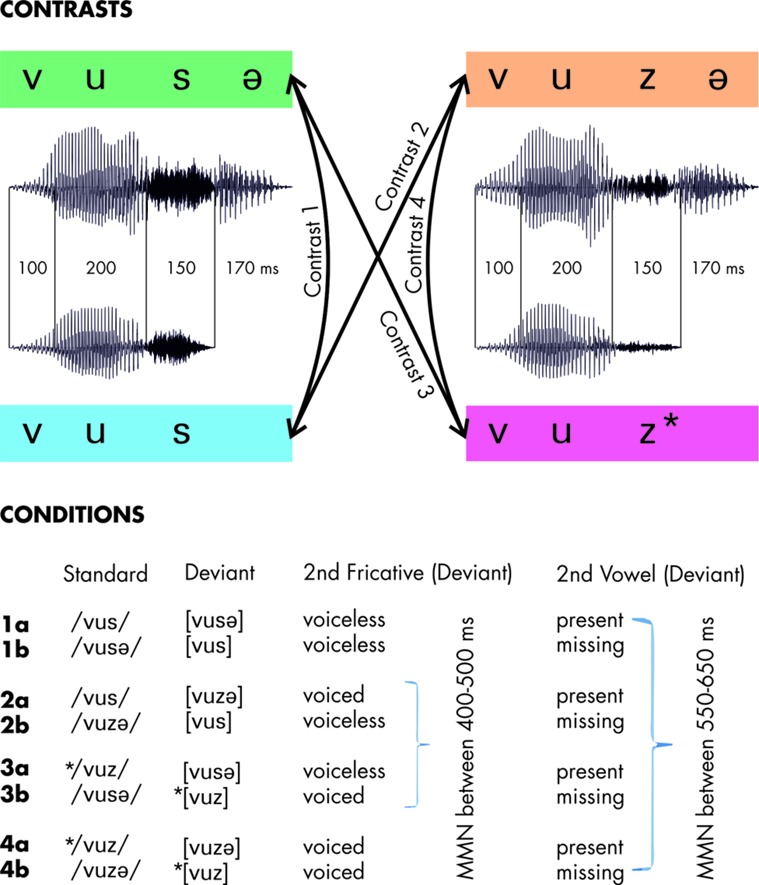
**Stimuli and design.** Representative waveforms depict the segmental and temporal structure of each of the four pseudowords. Arrays indicate the employed pair-wise oddball contrasts. The experimental conditions are listed below stating the segmental deviation criteria between the respective standard and deviant along with the time-ranges in which Mismatch Negativity (MMN) responses were expected in the difference waves. The asterisk * indicates ill-formedness.

The experiment reported here addressed German FD in pre-attentive phonological processing. The stimuli employed were [vus], ^∗^[vuz], [vusə], and [vuzə] as depicted in **Figure 1**. We concentrated on four pair-wise contrasts each of which was employed twice with reversed roles of standard and deviant in the stimulations, resulting in a total of eight experimental conditions. As explained above, we marked the standard stimuli of the experimental conditions with /./ and the deviants with [.]. Our expectations were based on the similarities of standard stimuli to abstract phonological lexical representations on the one hand, and deviants to phonetic surface representations that are close to the acoustic input on the other hand (Näätänen, 2001; Eulitz and Lahiri, 2004).

Contrast 1 is what would be an alternation in German for an underlying voiceless /s/. There is no change in voicing of the fricative. Hence, we expected MMN elicitation both for the additional vowel in 1a and for the missing vowel in 1b.

In contrast 2, stimuli differ with respect to the voicing of the second fricative. In condition 2a, this change is phonologically unmotivated. Furthermore, the deviant differs due to its additional vowel. Here, we expected an MMN to be elicited by each of these changes. Run the other way around, as in condition 2b, the whole contrast between standard and deviant could be interpreted as an alternation in German for an underlying voiced /z/ in /vuzə/, with FD in [vus]. While the phonetic differences were the same in 2a and 2b, we expected the respective MMN patterns to reflect that standard and deviant were phonologically related due to FD in 2b but not in 2a.

Contrasts 3 and 4 employ the ill-formed stimulus ^∗^[vuz]. It is ill-formed because FD would obligatorily turn it into [vus] in German. In contrast 3, the deviant enters into an unmotivated voicing alternation of the second fricative in both conditions. In addition, the stimuli differed with respect to the presence or absence of the final vowel. Consequently, we expected to find MMN for each of these segmental differences in both conditions. Furthermore, we were interested in whether we would find an additional effect attributable to the ill-formedness of ^∗^[vuz] when being presented as deviant. As the phonological violation ^∗^[vuz] coincided with the absence of the second vowel, we expected these mismatch responses to overlay, as reflected by a larger MMN amplitude in condition 3b compared to 1b.

In contrast 4, there is no change in voicing with respect to the second fricative, so no effects were expected in any corresponding time window. With respect to the difference in the final vowel, we were interested in whether condition 4a would show reduced MMN compared to the remaining a-conditions; this may be expected as it would reflect a remedy of the violation of FD in the standard ^∗^[vuz]. In condition 4b, we again expect superimposed mismatch effects due to the ill-formedness of the deviant ^∗^[vuz] and due to the missing second vowel as in condition 3b.

## MATERIALS AND METHODS

### PARTICIPANTS

Sixteen volunteers participated in the study (four male; median age was 26 years, range from 22 to 33), all of them right-handed and monolingual native speakers of German. Handedness was assessed using an inventory adopted from Oldfield (1971). All participants reported normal auditory and normal or corrected-to-normal visual acuity and no neurological, psychiatric, or other medical problems. They gave informed written consent. The study conformed to The Code of Ethics of the World Medical Association (2013, Declaration of Helsinki).

### MATERIALS

As described, four pseudowords were used as stimuli: [vus], [vusə], ^∗^[vuz], and [vuzə]. The stimuli are phonotactically well-formed in German, except for the non-word ^∗^[vuz], which fails to have undergone FD. The stimuli [vus], [vusə], ^∗^[vuz], and [vuzə] were articulated numerous times by a professional male speaker with a fundamental frequency (F_0_) of about 100 Hz, and digitally recorded with a 48 kHz sampling rate and a 16 bit resolution using a RME Fireface 800 recording device (Audio AG, Haimhausen, Germany) and a Neumann U87 Microphone (Georg Neumann GmbH, Berlin, Germany).

Stimulus preparation for ERP-experiments on speech processing is always a compromise. The point is to control for lower-level acoustic stimulus characteristics in order to avoid confounds with higher-level linguistic factors while on the same time keeping the stimuli as natural as possible and avoiding artifacts caused by manipulation. To assure some acoustic variability of the stimulus material, we selected 5 different utterances of each pseudoword resulting in a set of 20 pseudoword stimuli in total (see Eulitz and Lahiri, 2004; Jacobsen et al., 2004; Steinberg et al., 2012). However, the conflicting methodological requirements mentioned above concern our study in a special way. The phonological issue under investigation (i.e., the voicing distinction between [s] and [z]) is also coded by the inherent durational differences both between the voided and voiceless fricatives and between the preceding vowels. Other sufficient voicing cues for fricative perception are the presence or absence of low frequency energy during the fricative in the acoustic signal and distinct F1 transitions on vowel-fricative and fricative-vowel boundaries. These cues are also highly reliable at least in intervocalic and final fricative position (as in our stimuli).

Based on these considerations we decided to normalize the segmental durations of the stimuli across contrasts and to base the voicing distinction only on spectral phonetic parameters. Durational normalization was performed using the time-domain pitch synchronous overlap add (TD-PSOLA) algorithm provided by Praat software (Boersma and Weenink, 2010). Segmental durations were equated by setting the initial fricative to 100 ms (mean original durations of [v] in ms: [vus] 129, [vusə] 119, [vuz] 102, [vuzə] 109), the full vowel to 200 ms (mean original durations of [u] in ms: [vus] 200, [vusə] 188, [vuz] 285, [vuzə] 203), the second fricative to 150 ms (mean original durations of [s] in ms: [vus] 329, [vusə] 177; of [z] in ms: [vuz] 259, [vuzə] 119) and the final vowel to 170 ms (mean original durations of schwa in ms: [vusə] 189, [vuzə] 167). Afterward, intensities were normalized using the root mean square (RMS) of the whole sound file.

Theoretically, the duration normalization bore two risks: first, originally voiced fricatives might be perceived as voiceless after the relative lengthening of the fricative and the shortening the preceding vowel. Second, the contrary effect might have occurred to the originally voiceless fricatives. However, our ERP data clearly indicate that a distinction in the fricative has been detected in both directions in contrasts 2 and 3 [see *Analysis of the voicing change in the fricatives (contrasts 2 and 3)*]. Nevertheless, we performed acoustic analyses after the manipulation procedures to ensure that sufficient phonetic information was left in the stimulus material coding the voicing distinction between the fricatives [v] and [s] and to test potential interactions with the syllabic position of the fricative. We tested both offset F1 transitions of the first vowel, and the first two spectral moments of the fricative.

Formant measures were taken from each single stimulus file as mean values within 20 ms analysis windows by using the linear prediction-based burg method (as implemented in Praat) with a pre-emphasis frequency of 50 Hz. F1 measures were taken from the mid part (190–210 ms) and from the final part of the vowel (280–300 ms) by automatically determining maximally two formants below 2000 Hz. F1-transitions were analyzed by means of a univariate mixed design analysis of variance (ANOVA) with the within-items factor TRANSITION (mid vowel/vowel offset) and the between-items factors FRICATIVE (voiceless/voiced) and SYLLABLE (mono-/bisyllabic). We found a main effect of the factor TRANSITION (*F*_1,16_ = 6.2; *p* = 0.024; $\eta_{p}^{2}$ = 0.279) and a significant interaction TRANSITION^∗^FRICATIVE (*F*_1,16_ = 5.9; *p* = 0.027; $\eta_{p}^{2}$ = 0.269). As expected from the literature, the first vowel formant showed a significantly falling pattern when preceding the voiced fricative (F1 mid vowel: 357 Hz/F1 vowel offset: 316 Hz; main effect TRANSITION in a broken down two-way ANOVA with TRANSITION and SYLLABLE: *F*_1,8_ = 13.8; *p* = 0.006; $\eta_{p}^{2}$ = 0.633) while the F1 transition remained steady-state when being followed by the voiceless fricative (F1 mid vowel: 356 Hz/F1 vowel offset: 355 Hz; no significant effects). Note that there was no significant effect by the factor SYLLABLE.

To analyze the spectral qualities of the fricatives, FFT power spectra were calculated using a 50 ms Hann window that was centered over the mid part of the fricative (350–400 ms). From these spectra, COG and standard deviation (SD) were obtained. The spectral measures of the fricatives were analyzed by means of a multivariate ANOVA (MANOVA) with the between-items factors FRICATIVE and SYLLABLE as described before. A significant main effect of FRICATIVE indicates spectral differences between [s] and [z] (Pillai’s trace = 0.532; *F*_2,15_ = 8.5; *p* = 0.003; $\eta_{p}^{2}$ = 0.532). The factor SYLLABLE did not show any significant effects. The univariate analyses revealed that voiceless fricatives were characterized by significantly higher COG frequencies ([s] 7712 Hz/[z] 5908 Hz: *F*_1,16_ = 10.9; *p* = 0.004; $\eta_{p}^{2}$ = 0.406), and lower SD ([s] 2094Hz/[z] 2730 Hz: *F*_1,16_ = 14.0; *p* = 0.002; $\eta_{p}^{2}$ = 0.466) compared to the voiced fricatives. Based on this we assumed that the voicing distinction in the stimulus material was sufficiently coded phonetically even though durational voicing cues had been neutralized by manipulation.

### EXPERIMENTAL DESIGN AND PROCEDURE

As described above, four different experimental contrasts were employed: [vus] vs. [vusə] (contrast 1), [vus] vs. [vuzə] (contrast 2), ^∗^[vuz] vs. [vusə] (contrast 3) and ^∗^[vuz] vs. [vuzə] (contrast 4). Each pair-wise contrast was presented twice in oddball sequences, both using one pseudoword as standard (85% of the trials = 1360 items) and the other as deviant and the other way around (reversed oddball-design), resulting in eight experimental conditions. Oddball sequences of 1600 trials in total were presented per condition, using all tokens of each pseudoword equally. Standard and deviant stimuli were delivered in pseudo-randomized order forcing at least two standards to be presented between successive deviants. Oddball conditions were then divided into two technical blocks each, resulting in a total of 16 stimulation blocks per participant. Sessions were split into two parts, so the second half of each condition was presented on a second day. Stimulus sequences were presented with a stimulus onset asynchrony randomly varying from 550 to 900 ms in units of 10 ms. The order of the experimental blocks was counterbalanced between participants. Participants were seated comfortably in a sound-attenuated and electrically shielded experimental chamber, and they were instructed to ignore the auditory stimulation while watching a self-selected silent subtitled movie. Stimuli were presented binaurally at 53 dB SPL through headphones (Sennheiser HD 25-1 II; Sennheiser electronic GmbH & Co. KG, Wedemark, Germany). Loudness was measured by means of an artificial head (artificial head HMS III.2; HEAD acoustics GmbH, Herzogenrath, Germany). All participants reported that they were able to ignore the auditory stimulation. Informal questioning of the participants revealed that they had perceived all stimulus types as speech sounds. A whole experimental session lasted approximately 180 min (plus additional time for electrode application and removal) including ten short breaks of about 2 min each.

### ELECTROPHYSIOLOGICAL RECORDINGS

The EEG (Ag/AgCl electrodes, Falk Minow Services, V-Amp EEG amplifier; Brain Products GmbH, Gilching, Germany) was recorded continuously from 26 standard scalp locations according to the extended 10–20 system (American Encephalographic Society, 1994; FP1, FP2, F7, F3, FZ, F4, F8, FC5, FC1, FCZ, FC2, FC6, C3, CZ, C4, CP5, CP1, CP2, CP6, P7, P3, Pz, P4, P8, O1, O2) and from the left and right mastoids. The reference electrode was placed on the tip of the nose, and an additional electrode placed at AFZ was used as ground during recording. Electroocular activity was recorded with two bipolar electrode pairs, the vertical electrooculogram (EOG) was obtained from the right eye by one supraorbital and one infraorbital electrode and the horizontal EOG from electrodes placed lateral to the outer canthi of both eyes. Impedances were kept below 10 kʊ. On-line band-pass filtering of the EEG and EOG signals was carried out using a 0.011 Hz high-pass and a 100 Hz low-pass filter. The signal was digitized with a 16 bit resolution at a sampling rate of 500 Hz.

### DATA ANALYSIS

Off-line signal processing was carried out using EEP 3.0 (ANT Neuro, Enschede, Netherlands). EEG-data were band-pass filtered with a finite impulse response filter: 4001 points, critical frequencies of 0.5 Hz (high-pass) and 15 Hz (low-pass; cf. Schröger, 2005). EEG epochs with a total length of 1050 ms, time-locked to the onset of the stimuli and including a 100 ms pre-stimulus baseline, were extracted and averaged separately for each stimulus probability (standard, deviant), for each pseudoword, and for each participant.

The ERP responses to the first five stimuli per block as well as to each standard stimulus immediately following a deviant were not included in the analysis. Epochs showing an amplitude change exceeding 100 μV at any of the recording channels were rejected. In the present study, an average of 15.1% (SD 6.2%) of the trials per participant was rejected prior to ERP computation. Grand-averages were subsequently computed from the individual-subject averages.

To quantify the full MMN amplitude, the scalp ERPs were re-referenced to the averaged signal recorded from the electrodes positioned over the left and right mastoids. This computation results in an integrated measure of the total neural activity underlying the auditory MMN (e.g., Schröger, 2005).

Deviant-minus-standard difference waveforms were calculated for each pseudoword per oddball condition by subtracting the ERPs elicited by the standard point by point from the ERPs elicited by the original deviant obtained from the same oddball condition, i.e., the MMN elicited by [vusə] in condition 1a was quantified as difference between the deviant ERP from [vusə] and the standard ERP from [vus]. We opted for original contrasts from the same block in order to prevent superimposing effects from the block context to affect our comparisons.

Deviance-related effects (as the MMN) were quantified by measuring the ERP amplitudes as mean voltages in a fixed analysis window of 40 ms (for the width of the analysis window, cf. Luck, 2005, pp. 234). These windows were adjusted a posteriori on the basis of the grand-averaged deviance-minus-standard difference waves (cf. Picton et al., 2000). We adjusted separate windows for each condition and for each deviation by identifying the peak latencies of any distinguishable negative-going deflection (averaged across F3, Fz, F4, C3, Cz, and C4 electrode positions) within *a priori* determined time ranges. First, any effect due to the voicing alternation in the second fricative was expected to occur between 400 and 500 ms post stimulus onset (note that this latency equals 100 to 200 ms after the onset of the differing fricatives). This voicing alternation only occurs in contrasts 2 and 3. Second, deviations due to the presence or absence of the second vowel were expected to affect processing within the time range of 550–650 ms post stimulus onset (i.e., 100–200 ms after the offset of the fricative/onset of the final vowel). In singular cases, additional earlier or later time windows were analyzed in an exploratory approach.

Statistical analyses were performed with SPSS (IBM SPSS Statistics 21). As the MMN is known to be maximal over frontal scalp areas (cf. Kujala et al., 2007), we decided to base our analyses only on the F-line positions by collapsing the ERPs obtained at F3, Fz, and F4 into one single measure. Separately for each analysis window, an overall univariate repeated-measures analysis of variance, henceforth ANOVA, was run including the within-subjects factors STIMULUS PROBABILITY (standard/deviant), CONTRAST (depends on the window), and VOWEL (additional vowel in the deviant is present/missing). Afterward, analyses were broken down if appropriate. Finally, comparisons between conditions relating to the hypotheses were performed using repeated-measures ANOVAs with the factors introduced above. Only significant main effects of the factor STIMULUS PROBABILITY and interactions with this factor were reported. The level of type 1 error was set to *p* < 0.05 and, in case of multiple *post hoc* comparisons, Bonferroni correction was applied. If the sphericity assumption was violated (indicated by the Mauchly test), the original degrees of freedom were provided along with the Greenhouse-Geisser-epsilon. Finally, partial eta-squared ($\eta_{p}^{2}$) effect sizes were given for all significant effects.

## RESULTS

**FIGURE 2:**
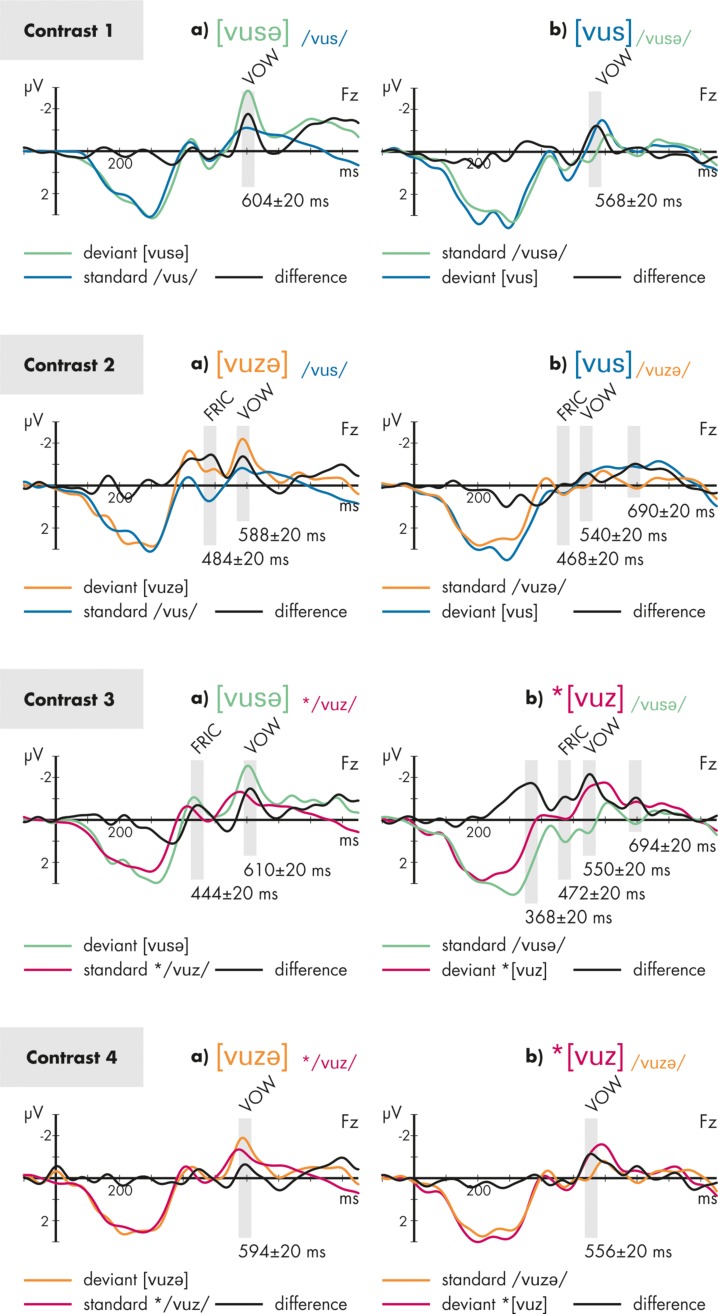
**Grand-averaged and re-referenced event-related potentials (ERPs) elicited by the stimuli of Contrasts 1–4 depicted separately for conditions a (left) and b (right) at FZ electrode site.** The color of the ERPs codes the stimulus that elicited that ERP. The black line represents the difference wave calculated for each condition. The gray bars indicate the time windows of statistical analyses. FRIC indicates that the marked time range is attributed to the voicing change in the second fricative, VOW indicates that the marked time range is attributed to the final vowel. The asterisk * indicates ill-formedness.

**FIGURE 3:**
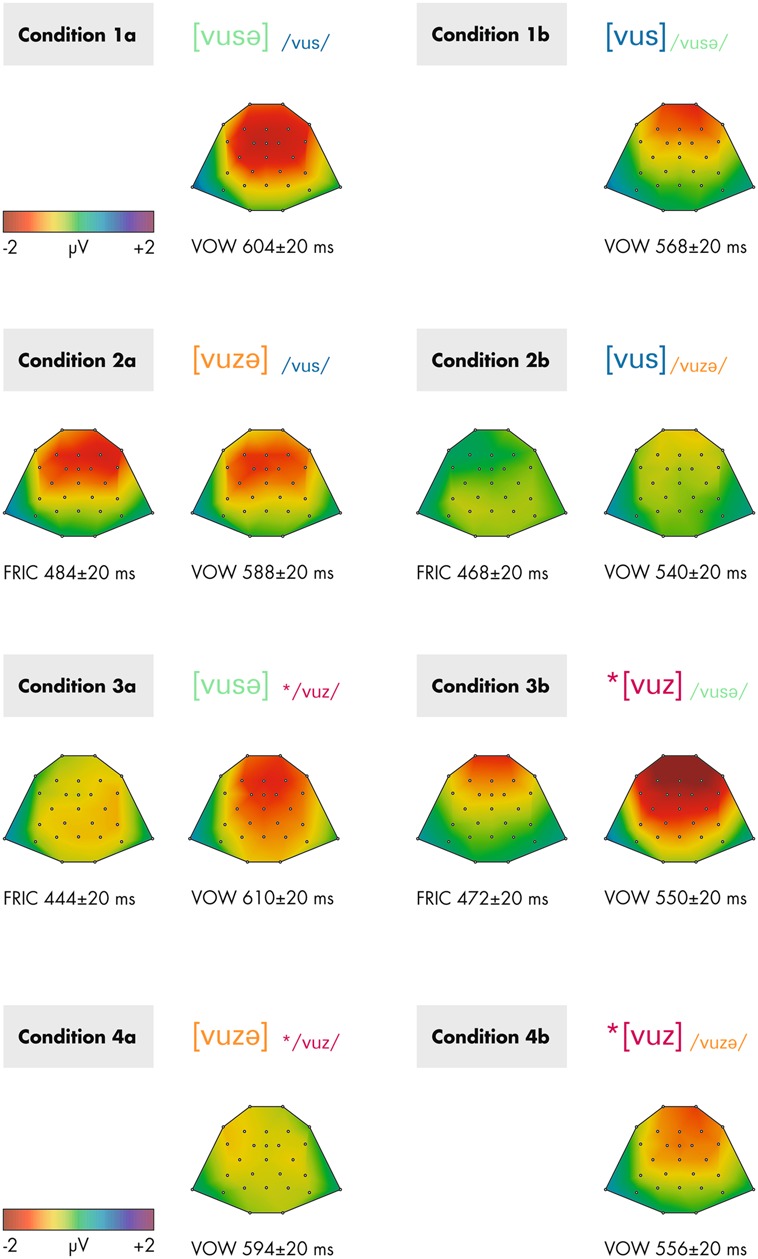
**Topographical maps of the analyzed deviance-minus-standard differences obtained from grand-averaged re-referenced data within the time windows stated below each map.** Depicted are contrasts 1–4 separately for conditions a (left) and b (right). FRIC indicates that the marked time range is attributed to the voicing change in the second fricative, VOW indicates that the marked time range is attributed to the final vowel. The asterisk * indicates ill-formedness.

The ERP results for all conditions are depicted in **Figure 2**. Also, this figure shows the respective analysis windows for each effect. The outcomes of the statistical analyses based on these windows are presented below separately for each analysis window. In **Figure 3**, topographical maps of the analyzed MMN effects are provided separately for each condition and time window.

### ANALYSIS OF THE VOICING CHANGE IN THE FRICATIVES (CONTRASTS 2 AND 3)

For the MMN responses to the fricatives (FRIC in **Figure 2**) the overall ANOVA with the factors STIMULUS PROBABILTIY (standard/deviant), VOWEL (additional/missing), and CONTRAST (2/3) revealed a significant main effect of the factor STIMULUS PROBABILITY (*F*_1,15_ = 17.9; *p* = 0.001; $\eta_{p}^{2}$ = 0.544), indicating the presence of an MMN across all conditions, and a significant interaction STIMULUS PROBABILITY^∗^VOWEL^∗^CONTRAST (*F*_1,15_ = 5.9; *p* = 0.028; $\eta_{p}^{2}$ = 0.284), indicating different amplitudes of the MMN responses across conditions. Broken-down analyses were calculated separately for each contrast: in contrast 2, the main effect for STIMULUS PROBABILITY (*F*_1,15_ = 9.1; *p* = 0.009; $\eta_{p}^{2}$ = 0.387), and also the interaction STIMULUS PROBABILITY^∗^VOWEL (*F*_1,15_ = 5.9; *p* = 0.028; $\eta_{p}^{2}$ = 0.282) were significant, the latter indicating a stronger MMN response in condition 2a compared to condition 2b. In contrast 3, only a significant main effect for STIMULUS PROBABILITY was obtained (*F*_1,15_ = 9.3; *p* = 0.008; $\eta_{p}^{2}$ = 0.384).

### ANALYSIS OF THE EFFECT DUE TO THE CHANGE IN THE FINAL VOWEL (ALL CONTRASTS)

For the MMN responses to the additional or missing vowel (VOW in **Figure 2**) the overall ANOVA with the factors STIMULUS PROBABILITY (standard/deviant), VOWEL (additional/missing), and CONTRAST (1/2/3/4) revealed a significant main effect for STIMULUS PROBABILITY (*F*_1,15_ = 25.0; *p* < 0.001; $\eta_{p}^{2}$ = 0.625), as well as significant interactions STIMULUS PROBABILITY^∗^CONTRAST (*F*_3,45_ = 4.0; *p* = 0.026; ε = 0.725; $\eta_{p}^{2}$ = 0.209) and STIMULUS PROBABILITY^∗^CONTRAST^∗^VOWEL (*F*_3,45_ = 3.1; *p* = 0.039; ε = 0.928; $\eta_{p}^{2}$ = 0.172). Next, analyses were broken down by the factor VOWEL. Comparing the MMN amplitudes for the a-conditions, only a significant main effect for STIMULUS PROBABILTY (*F*_1,15_ = 9.3; *p* = 0.008; $\eta_{p}^{2}$ = 0.382) was found, but no interaction with this factor. For the b-conditions, the main effect STIMULUS PROBABILITY (*F*_1,15_ = 21.1; *p* < 0.001; $\eta_{p}^{2}$ = 0.585) and the interaction STIMULUS PROBABILITY^∗^CONTRAST (*F*_3,45_ = 6.2; *p* = 0.002; ε = 0.880; $\eta_{p}^{2}$ = 0.292) were significant. This interaction indicates differences in MMN amplitudes due to the missing final vowel across the contrasts. We *a priori* were only interested in potential differences between conditions 1b and 3b, both sharing the same legal standard /vusə/. A broken-down ANOVA with STIMULUS PROBABILITY and CONTRAST (1/3) revealed a significant main effect for STIMULUS PROBABILITY (*F*_1,15_ = 35.4; *p* < 0.001; $\eta_{p}^{2}$ = 0.703), and a significant interaction between both factors (*F*_1,15_ = 5.1; *p* = 0.039; $\eta_{p}^{2}$ = 0.252), indicating stronger MMN amplitudes for 3b compared to 1b.

### EXPLORATIVE ANALYSES OF EARLIER AND LATER EFFECTS (CONTRASTS 2 AND 3)

In conditions 2b and 3b, unexpected deviance-related effects were found in a time range later than 650 ms post stimulus onset. These effects were analyzed as described above: a significant main effect of STIMULUS PROBABILTIY (*F*_1,15_ = 19.5; *p* = 0.001; $\eta_{p}^{2}$ = 0.565) was found but no interactions with this factor. Because of its latency, it seems possible to us that this effect reflects morphological processing (see Royle et al., 2010). This is conceivable if the additional vowel is processed as a morphological suffix.

Furthermore, a strong deviance-related effect was observed in condition 3b that appeared in an unexpected early time range before 400 ms, i.e., before the onset of the deviating fricative. Because of its latency, this effect seemed to be temporally related to the later part of the first vowel [u]. This effect was compared with a corresponding time window in condition 1b (360 ± 20 ms) that shared the legal standard stimulus /vusə/. A significant main effect STIMULUS PROBABILITY (*F*_1,15_ = 39.1; *p* < 0.001; $\eta_{p}^{2}$ = 0.722) was found as well as a significant interaction STIMULUS PROBABILITY^∗^CONTRAST (*F*_1,15_ = 9.0; *p* = 0.009; $\eta_{p}^{2}$ = 0.374), indicating a stronger deviance-related response in 3b compared to 1b.

## DISCUSSION

### REMARKS ON CONTRAST 1

Our statistical assessment employed condition 1b as a comparison condition for condition 3b (see *Evidence for the relevance of final devoicing in condition 3*). However, contrast 1 (pairing the legal stimuli [vusə] and [vus] with no voicing change) is here also briefly considered on its own. Visual inspection of the difference waves for contrast 1 in **Figure 2** shows distinct MMN responses that are attributable to the presence vs. absence of the final vowel, but no further effects, in particular no effects between 450 and 550 ms where differences attributable to the fricative would occur. This provides some assurance that effects attributed to fricatives in other conditions were not general consequences of our stimulus contrasts in which stimuli with and without a final vowel are compared. There is, for example, a distinction in syllabification. The a-conditions are syllabified like 1a: [vu.se] while the b-conditions are single syllables like 1b: [vus]. This distinction could in principle have phonetic correlates in regard to the extent of coarticulation of the [s/z] with the preceding vowel. Recall that the phonetic analysis of the stimuli did not detect any such differences. Condition 1 suggests that such differences, if they exist after all, also did not lead to observable effects in the difference wave.

### EVIDENCE FOR THE RELEVANCE OF FINAL DEVOICING IN CONTRAST 2

The following sketch shows condition 2b next to condition 2a. We included a dot to mark the syllable boundary in [vu.zə].


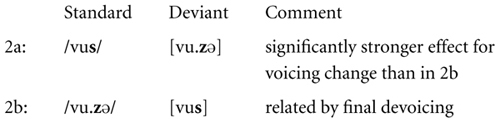


There is a significant difference between conditions 2a and 2b in the processing correlates of the voicing change in the fricative. The MMN effect due to the voicing mismatch in condition 2a was absent in condition 2b, where the voicing change was motivated by FD. This significant difference between conditions 2a and 2b is here interpreted as evidence for the relevance of FD in pre-attentive processing.

### REMARKS ABOUT REACTIONS TO THE FINAL VOWEL IN CONDITION 2b

We turn to some remarks about the MMN response due to the additional/missing vowel in conditions 2a and 2b. The plots in **Figure 2** suggest that the response attributable to the missing final vowel in the deviant of condition 2b was also reduced. We here want to comment this impression for the benefit of possible future experiments that might investigate such an effect more specifically. The observation suggests that the expectation of any upcoming auditory event, which is violated in the deviant and shown by the MMN, is not limited to the expectation of just another standard stimulus. It seems, instead, that this expectation can be modulated by what is found earlier in the deviant. The system seems to have related /vuzə/ and [vus] by FD. If the system possesses knowledge of the environment of FD, it will then expect the absence of a vowel following [vus], since FD would not have applied in the presence of a following vowel. (Similar expectations could also be modulated by phonetic factors that might allow the anticipation of the absence of a final vowel. However, the reduced MMN response to the missing final vowel seems to be specific to condition 2b, where FD has applied.) It is also possible, then, that the standard /vuz+ə/ and the deviant [vus] were processed as morphologically related by the omission of an inflectional element [ə] in the deviant, with phonological adjustment due to FD. It seems conceivable that this was related to the late deviance-related effect that was observed about 250 ms after the missing vowel had become detectable.

We note that we have argued (Jacobsen et al., 2013) against the assumption of successive MMN responses in case of mismatching monosyllabic vowel-consonant sequences, where both the vowel and the consonant differed. However, the case at hand is different in an important aspect: the second deviation in the present contrast pairs, namely the missing or additional final vowel in contrasts 2 and 3, did not just involve a distinct sound, but established a distinction in syllable structure between standard and deviant. By this, the present stimulus contrasts were clearly different not just at the segmental but also at suprasegmental representation levels.

### EVIDENCE FOR THE RELEVANCE OF FINAL DEVOICING IN CONDITION 3

It was seen in the presentation of the results that condition 3b and condition 1b both have MMN responses attributable to the missing vowel, and that both effects furthermore differ significantly in strength. This is illustrated in the following sketch.


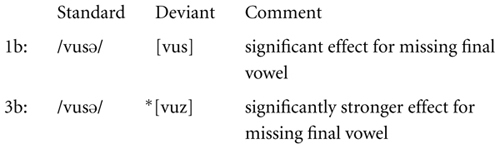


It was suggested that this is evidence for a superposed effect of the ill-formedness of the deviant ^∗^[vuz] in condition 3b, which becomes manifest in the signal simultaneously with the absence of the final vowel. This distinction provides further evidence for the relevance of FD in pre-attentive processing.

### REMARKS ON REACTIONS TO THE FIRST VOWEL IN CONDITION 3

The comparison between conditions 1b and 3b is repeated in the following, this time highlighting a significant distinction that was found post hoc: condition 3b showed an effect at the time at which the second part of the vowel [u] is expected to be processed. The distinction to 1b was seen to be significant.


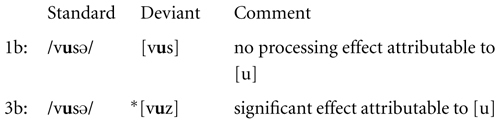


This effect in 3b may be related to the anticipation of [z] during the vowel [u] due to coarticulatory cues. It is furthermore possible that phonetic factors allowed an early prediction of the syllable structure. The system might have noticed in ^∗^[vuz] during the vowel that there would be an upcoming voiced fricative within the same syllable, in violation of FD. If so, the early strong MMN effect before 400 ms in condition 3b might already be a first electrophysiological response to the ill-formedness of the deviant.

## SUMMARY

In summary, we have found two pieces of evidence for the role of FD in pre-attentive processing. While condition 2a, [vuzə]_/vus/_, showed mismatch effects due to the voicing change in the fricative, these are significantly reduced (and in fact absent) in condition 2b, [vus]_/vuzəs/_, in which the two forms can be related by FD. In condition 3b, ^∗^[vuz]_/vusəs/_, an overlaid effect of the violation of FD in the deviant ^∗^[vuz] was found.

An interesting aspect of our findings is that they provide evidence that syllable-related lexical phenomena such as FD are already taken into account by the processing system in an early pre-attentive stage. This point is new insofar the only previous study we are aware of that showed the processing relevance of a syllable-related process is Dehaene-Lambertz et al. (2000), which did not employ a pre-attentive protocol.

## Conflict of Interest Statement

The authors declare that the research was conducted in the absence of any commercial or financial relationships that could be construed as a potential conflict of interest.
